# COVID-19 Treatment Guidelines: Do They Really Reflect Best Medical Practices to Manage the Pandemic?

**DOI:** 10.3390/idr13020029

**Published:** 2021-04-01

**Authors:** Feras Jirjees, Ali K Saad, Zahraa Al Hano, Taher Hatahet, Hala Al Obaidi, Yahya H Dallal Bashi

**Affiliations:** 1 College of Pharmacy, University of Sharjah, Sharjah, United Arab Emirates; zalhano@sharjah.ac.ae; 2 College of Medicine and Health Sciences, United Arab Emirates University, Alain, United Arab Emirates; 3 School of Pharmacy, Queens University Belfast, Belfast, UK; T.Hatahet@qub.ac.uk (T.H.); ydallalbashi01@qub.ac.uk (Y.H.D.B.); 4 Queen’s University Belfast Joint College (CQC), China Medical University, No. 77 Puhe Road, Shenyang North New Area, Shenyang 110122, China; 5 Pharmacy Department, City University College of Ajman, Ajman, United Arab Emirates; o.hala@cuca.ae

**Keywords:** COVID-19 treatment guidelines, therapeutics guidelines, medications, pharmacological management

## Abstract

SARS-CoV-2 (COVID-19) has been changing the world since December 2019. A comprehensive search into many COVID-19 treatment guidelines was conducted and reported in this article. This is a review paper to probe differences in COVID-19 managing strategies and explore the most common treatment plans among countries. Published guidelines from 23 countries and three references guidelines—until the end of 2020—were included in this article. The majority of COVID-19 treatment options were reported in this review and it includes antiviral drugs, antimalarial drugs, antibiotics, corticosteroids, immunotherapy, anticoagulants, and other pharmacological treatment. The presence of such information from different countries in a single comprehensive review article could help in understanding and speculation of variation in the recommended treatment in each country. This might be related to the cost of medications, the access to the medications, availability of medication that could potentially be useful in managing COVID-19 cases, and the availability/capacity of healthcare facilities. Finally, although there are various treatment groups listed in the published therapeutic guidelines worldwide, unfortunately, there is no evidence for effectiveness of most of these medications in reducing the COVID-19 mortality curve over more than one year of this global pandemic.

## 1. Introduction

The emerging of SARS-CoV-2 (COVID-19) virus to all parts of the world within three months from the first case recognized in Wuhan, China, proves the contagious nature of this virus. Then, the dramatic increase in infected cases to over 83 million people, and over 1.8 million death worldwide in the first year of this pandemic [1], demonstrated the severity of disease, where all health systems worldwide were not able to control such high mortality rate. Changes in the economy, revelations of social differences among societies, and overwhelming health care systems put governments under huge pressure to fight against the spread of this virus [2].

COVID-19 is an RNA airborne virus infection leading to varied morbidities and mortalities. Some COVID-19 patients are asymptomatic, while others develop a severe pneumonia ending with respiratory failure. This disease begins with a first phase of viral replication followed by a second phase of inflammatory response [3]. There is no concrete evidence, until today, on the effectiveness and safety of the treatment of COVID-19 patients. Medications administered in the inpatient or ICU settings are based on previous efficacy results in treating, malaria, rheumatoid arthritis, Middle East respiratory syndrome (MERS), severe acute respiratory syndrome (SARS), Ebola, influenza, autoimmune diseases, respiratory infections, secondary bacterial infection, acute respiratory distress syndrome (ARDS), sepsis, cytokine storm, and organ or multi-organ failure. Up to date, some COIVD-19 vaccines had been approved and reached the market in late 2020.

With the aim of reviewing the most up to date COVID-19 management options, therapeutic guidelines of many countries worldwide were investigated. This paper presents and discusses medications and supportive therapies to treat patients with COVID-19 infection, aiming to probe, explore, and summarize various COVID-19 therapeutic protocols (Table 1). This article can be extremely useful for practitioners and health care authorities.

## 2. Therapeutics Guidelines

Many treatment guidelines have been published on management of patients infected with COVID-19. The recommendations are based on expert opinions and some scientific evidence. Nevertheless, there is no definitive agreed-upon pharmacological management of COVID-19 and most recommendations rely on the supportive treatment. Additionally, encouraging results from COVID-19 related clinical trials of remdesivir and dexamethasone had led to introduce these medications into the Food and Drug Administration (FDA), European Medicines Agency (EMA), and several national guidelines [5,6,7,8,9,11,12,13,14,16,17,18,19,20,24,25,26,27,28,29,31].

Due to the nature of the situation and its novelty, most of the medications used in these national guidelines have not been well studied against COVID-19 infection. Furthermore, hydroxychloroquine (HCQ), chloroquine (CQ), and lopinavir/ritonavir have been found to be ineffective according to large clinical trials; however, these medications have not been removed from several COVID-19 treatment guidelines. Surprisingly, recent results of the Solidarity trial conducted in 30 countries worldwide revealed that many regimens have minor or no effect for treatment of hospitalized COVID-19 patients including remdesivir [34]. Therefore, the antiviral and immunotherapy agents required further testing of their efficacy in a larger population before any beneficial effect could be confidently announced [35,36,37,38].

COVID-19 treatment guidelines across countries have many similarities such as treatment with the main four groups of medications: antiviral, antimalarial, systematic corticosteroids, and antibiotics, with variations of drugs, dosages, and durations. These variations further stress the inadequate evidence to support any pharmacological treatment.

Importantly, the cost of a COVID-19 treatment course is not available in these guidelines. Prices of antimalarial and corticosteroid therapy can be affordable for many countries worldwide. However, antiviral and immunomodulators are much more expensive, and this could hinder their availability to all patients in need for such therapies especially in low-income countries. For example, the cost of remdesivir is around 552 USD/per 100 mg powder for injection (3312–6072 USD for the complete adult treatment course) and Kaletra^®^ (Lopinavir/ Ritonavir) costs around 544 USD/per 160 mL of (400 mg/100 mg/5 mL) (554 USD for the complete adult treatment course), Actemra^®^ (Tocilizumab) costs about 490 USD per 4 mL of 20 mg/mL injection solution (2450–4900 USD for complete course), and Rebif^®^ (Interferon beta 1a) is 9273.69 USD per 6 mL of 44 mcg/0.5 mL (2318–3803 USD for the complete adult treatment course) [39]. In general, these medications cannot be adapted at a national scale in many developing countries.

## 3. Antiviral Drugs

Typically, antiviral drugs directly target the infecting pathogen to halt its development. Some agents are specific for certain types of viruses, others have a broad-spectrum of activity. Nine antiviral drugs (monotherapy or in combination) were documented in the national guidelines for treatment of COVID-19 worldwide. Each of these drugs have shown antiviral effects against one of the following viruses that cause MERS, SARS, Ebola, influenza, or human immunodeficiency virus (HIV) [40]. These drugs have various mechanism of actions; therefore, they have diverse efficacy and safety profiles. Despite many clinical trials being conducted to assess the efficacy of these antivirals against COVID-19, only one antiviral drug (remdesivir) has been approved for use for the treatment of hospitalized COVID-19 patients [41].

Generally, the antiviral drugs use recommendation is included in the therapeutic guidelines listed in Table 1. In these COVID-19 management guidelines, the antiviral drugs are not commonly used for severe cases but can be considered as an additional or an alternative treatment in case there is an issue with the use of the first-choice drug. Apart from remdesivir, the Netherlands’ guideline does not recommend the use of any antiviral drugs as a first-line treatment for COVID-19 cases [20]. In Africa, Nigeria’s guideline prohibits the administration of all antivirals unless in a clinical trial setting [33]. In Asia, the concomitant use of three or more antiviral drugs is not advised according to Chinese recommendations [26,27].

The use of these antiviral agents in managing COVID-19 cases is debatable among countries as stated in their varied COVID-19 therapeutic guidelines due to their availability and efficacy.

### 3.1. Remdesivir

Remdesivir is the only antiviral drug approved by the FDA for treatment of COVID-19 [41]. It can be used for severe cases as recently supported by the findings of numerous clinical trials which have been conducted to evaluate its efficacy in COVID-19 patients [42,43]. Preliminary findings of the largest multicenter clinical trial indicated that remdesivir can shorten the recovery time with the aid of oxygen supplementation. However, a monotherapy treatment is unadvised due to high mortality rate in the treatment group [19].

Remdesivir is administered intravenously (IV) as a loading dose of 200 mg for 30 min then maintained by 100 mg IV daily for 4 to 9 days. Singapore’s treatment guideline recommended early use of remdesivir to minimize viral load and lung damage [29].

The Belgian guideline limited the use of this drug to those who are not administering other investigational treatment in clinical trials including HCQ/CQ and recommended to closely monitor the drug when given with other drugs [14]. Nigeria, also, does not recommend its use unless in a clinical trial setting [33]. Although no drug–drug interaction studies have been conducted for remdesivir, the Gilead Company is not recommending the use of remdesivir in combination with CYP450 inducers such as rifampin [44].

In a recent update, the World Health Organization (WHO) has issued a conditional recommendation against the use of remdesivir in hospitalized patients, regardless of disease severity. This is explained by the current lack of evidence that remdesivir improves survival and other outcomes in these patients [45].

Remdesivir is approved for treatment of hospitalized adult patients with COVID-19 and pediatric patients above 12 years of age or weighing over 40 kg [41]. There is no clear evidence yet on the efficacy of remdesivir treatment for pregnant and lactating mothers. Looking into remdesivir use in pregnant, lactating mothers and the pediatric sup-population, the drug was not recommended for children before COVID-19 pandemic [44]. Remdesivir has been recommended for the compassionate use (pregnant or children below 12 years) or in severe confirmed cases by most European countries (EMA program), and in other national treatment guidelines [9,11,12,13,29,31]. Emergency use authorization (EUA) permitted the use of remdesivir on pediatric patients below 12 years old or between 3.5 and 40 kg body weights [5].

The Indian clinical management protocol issued in June 2020 recommends the use of remdesivir, after the EUA, in moderate cases requiring oxygen supplementation but considers pregnancy, lactation, and young age (less than 12 years old) as contraindications [5].

In Spain, the COVID-19 treatment guideline has clear information on dosing in children weighing more than 3.5 kg [21]. The pharmacokinetics and safety of remdesivir in children are currently under evaluation in many clinical trials worldwide [46].

There is no study yet to ensure remdesivir safety and proper dosing in patients with liver and kidney dysfunction. The drug is contraindicated in case of renal (CrCl < 30 mL/min) and hepatic dysfunction [30]. Several treatment guidelines clearly address this issue in the drug contraindication section [14,17,21,24,29,31]. Finally, Italian guideline attachment for extended access protocol by Gilead stated that no dosage modification is needed in patients with liver and kidney malfunction [17].

### 3.2. Lopinavir/Ritonavir

The utilization of this antiviral combination in COVID-19 patients is based on retrospective evidence of its effectiveness against MERS and SARS [47,48]. However, the outcomes of a recent clinical trial (LOTUS trial) indicated that this combination adds no beneficial value compared to standard care in COVID-19 patients [49]. Eventually, the WHO stopped the lopinavir/ritonavir (LPV/r) study due to the little or no reduction in hospitalized patients’ mortality compared to the standard care [50]. For the treatment of COVID-19, the common, recommended dosage is 400 mg of lopinavir with 100 mg of ritonavir twice daily for 14 days [49].

In some national guidelines, this combination is mainly used in cases where HCQ/CQ is contraindicated or unavailable [14,16]. The Italian treatment guideline enabled the use of this combination in mild cases (but suspended its use in July 2020 update) [18]. The National Institutes of Health (NIH) guideline does not recommend the use of LPV/r or any other protease inhibitor for COVID-19 patients [9]. The Nigerian guideline does not recommend its use unless in a clinical trial setting [33]. In Thailand and Egypt guidelines, LPV/r is a component of a combination treatment consisting of CQ or HCQ, azithromycin, and/or favipiravir depending on the severity of the symptoms [10,25].

The LPV/r is a safe option in pregnancy. However, an issue was raised in Ireland guideline related to infected pregnant patients with diabetes, as these patients advised to closely monitor glucose when using these drugs [16]. Furthermore, fetal harm cannot be ruled out as stated by Saudi Arabia’s treatment guideline [12].

LPV/r combination can potentiate QT segment prolongation and negatively influence heart rhythm coordination. Therefore, it should be avoided in patients with congenital long QT syndrome or hypokalemia and in simultaneous use with other QT prolonging agents. Furthermore, the LPV/r combination should be avoided with drugs that increase QT interval such as HCQ, CQ, azithromycin, and fluoroquinolones; this was highlighted in many guidelines [16,17,25,27,29]. Both agents of the LPV/r combination inhibit CYP3A4; therefore, drugs that substrate this enzyme should be avoided or adjusted in dosing to prevent toxicity. The LRV/r–drug interaction profile is clearly described in the published guidelines [16,21,28,29]. The LPV/r combination is contraindicated in patients with renal failure and/or hepatic impairment as indicated in Saudi Arabia guideline [12].

### 3.3. Darunavir/Cobicistat (DRV/c)

This antiviral combination is indicated in two guidelines (Italy and China) as a safer alternative when the LPV/r combination is contraindicated [17,28]. It is given at a dose of 800 mg darunavir with 150 mg cobicistat daily for 5–7 days. The Italian guideline suspended the off-label use of these drugs in July 2020 update along with LPV/r [17]. This can be explained by the lack of information about the combination on COVID-19 virus. There is no evidence found on the effectiveness of darunavir in managing COVID-19 yet [51]. Furthermore, this combination has not proven any increase in the proportion of negative polymerase chain reaction (PCR) conversion within 5 days treatment in patients with mild symptoms in China [52]. The last point of consideration is related to darunavir drug-drug interaction profile, specifically with statin drugs (prescribed for dyslipidemia) occurring upon long term co-administration [53].

### 3.4. Favipiravir

Favipiravir has been approved for the treatment of influenza in Japan in 2014 [54]. Focusing on the COVID-19 virus, a randomized clinical trial performed in China for treating infected patients found that favipiravir can reduce the time to fever and cough relief [46]. For COVID-19 management, favipiravir has commenced with phase 2 and 3 clinical trials in several countries: Italy (NCT04336904), Russia (NCT04434248), USA (NCT04358549), Japan, India, and Saudi Arabia (NCT04392973). Favipiravir is used in a loading dose of 1600 mg twice daily followed by 600 mg maintenance twice daily for five days in managing COVID-19 cases.

As an empirical treatment, the guidelines of Thailand, UAE, and Malaysia enabled the use of this drug with or without HCQ/CQ in severe cases [13,25,30], while it is not recommended in Nigeria unless in a clinical trial setting [33].

Favipiravir is absolutely contraindicated in pregnancy. Moreover, favipiravir can distribute to the sperms; therefore, it is necessary to check its use in male patients [55].

Regarding special precautions with patients’ subgroups such as children and elderly, pediatric dosing on patients weighing 10 kg and above is clearly described in the Saudi Arabia treatment guideline [12]. Favipiravir can cause renal toxicity especially in the elderly patients (with risk factors for chronic kidney disease). The close monitoring with dose or duration adjustment is always needed [13]. 

### 3.5. Umifenovir

In China, umifenovir is a licensed drug used in treating influenza virus a long time ago [55]. In a retrospective Chinese study, umifenovir was unable to improve the prognosis of COVID-19 in non-intensive care units [56]. In addition, a systematic review and a meta-analysis conducted by Huang et al. (2020) concluded there is no evidence to support the use of umifenovir for improving outcomes in patients with COVID-19 infection [57]. The Chinese guideline recommended a combination treatment with LPV/r for COVID-19 patients. Umifenovir is prescribed as 200 mg four times daily for 5 to 7 days for COVID-19 patients. Nigeria does not recommend its use unless in a clinical trial setting [33]. It should be used with caution in patients with severe renal dysfunction or a sinus node disease [27].

### 3.6. Ribavirin

Ribavirin has a broad-spectrum activity against several RNA and DNA viruses. Earlier evidence with MERS-CoV supported its potential activity against COVID-19 virus [58]. The drug is used as 500 mg IV infusion dose 2 to 3 times daily for 10 days (in China) or 400 mg orally twice daily for 14 days in Saudi Arabia and Singapore or 7 days in the UAE [12,13,28,29]. Ribavirin is used in a double therapy protocol with LPV/r or interferon alpha as recommended by the pharmaceutical Association in China [27].

An early triple-therapy protocol including ribavirin, LPV/r, and subcutaneous Interferon beta-1b was more effective than LPV/r alone in treating patients with mild to moderate COVID-19 symptoms as concluded by the results of a randomized, multicenter clinical trial in Hong Kong [59]. This triple-therapy protocol is recommended early (within 7 days of the onset of COVID-19 symptoms) in Saudi Arabia, Singapore, and the Chinese National Health Commission guidelines [12,28,29]. In Saudi Arabia, this combination is used for moderate to severe cases but not in critically ill patients [12]. Conversely, it is only recommended in critical cases in the UAE [13]. In Singapore, this treatment is a third-line option after remdesivir and the convalescent plasma therapy in severe cases or once oxygen supplementation is required [29].

Ribavirin has a negative impact on hemoglobin concentrations [58]. This adverse effect is particularly unfavorable in the treatment of a disease characterized by degrees of respiratory malfunction such as COVID-19 Saudi Arabia and the UAE guidelines pointed out an anemic risk in their contraindication and side effects sections [12,13]. This anemia could worsen any underlying cardiac disease, and causing myocardial infarction; therefore, electroencephalogram (EEG) monitoring is advised by the Singapore guideline [29]. Furthermore, ribavirin should be avoided in the senior population according to the Chinese Pharmaceutical Association [27].

When looking into the drug-associated adverse reactions and special precautions, ribavirin is found to be teratogenic, therefore it should be avoided in pregnancy. Both male and female patients should be advised to avoid planning for a baby for 6 months after taking the drug as stated in Chinese Pharmaceutical Association, Singapore, and Saudi Arabia guidelines [12,27,29]. Ribavirin is mainly excreted by the kidneys and accumulation could occur in case of renal insufficiency [60]. It is contraindicated in renal impairment (CrCl < 50 mL/min) as suggested by guidelines of Saudi Arabia, UAE, and Chinese Pharmaceutical Association [12,13,28]. The renal function should be monitored prior to use of ribavirin according to Singapore guideline [29].

### 3.7. Oseltamivir

Oseltamivir is a selective neuraminidase inhibitor which used to treat influenza (A and B), and the dosage prescribed as a 75 mg dose twice daily for 5 days [61]. Oseltamivir is indicated for treatment of influenza virus in hospitalized young children, patients over the age of 65 years, and patients with other significant health problems, or pregnant women. This drug may lower the duration of fever of the outpatients when it given shortly after having COVID-19 symptoms as per Chiba study [62].

The Egyptian health authorities enabled the use of oseltamivir with all cases of COVID-19 [10]. Oseltamivir interacts with a low number of drugs bringing its advantageous use with chronic disease patients.

## 4. Antimalarial Drugs

Hydroxychloroquine (HCQ) and chloroquine (CQ) originally are antimalarial drugs prescribed to treat some autoimmune diseases. At the beginning of the COVID-19 pandemic (early 2020), both the drugs were recommended to be used in many COVID-19 treatment guidelines worldwide. Currently (late 2020), HCQ and CQ are not highly used or not recommended for the treatment of COVID-19 patients in many guidelines [9,11,18,22,24,29,31,32].

The WHO paused the use of HCQ in the intervention arm (and HCQ discontinued in July 2020 as a result of clinical trial evidence) of the SOLIDARITY study in May 2020 [34]. This was attributed to a study published by the Lancet journal which concluded that these drugs have no clinical benefit in managing COVID-19 patients [63], but rather carry an increased risk of the life-threating ventricular arrhythmias [64,65,66]. However, the study was later retracted due to methodological and data integrity concerns [63].

The use of HCQ and CQ for COVID-19 patients were first highlighted in an open-label non-randomized French clinical trial [67]; however, they have not been included in the treatment guideline of France outside clinical trials [15]. On the other side, these drugs were available in most treatment guidelines and numerous national and international clinical trials were conducted to evaluate the efficacy and safety of these drugs in treating COVID-19 patients.

The discontinuation of the HCQ arm in the RECOVERY study [68], due to the lack of clinical benefits of the drug in hospitalized COVID-19 patients, has imposed changes on the recommendations in different guidelines to prevent using these agents outside the scope of clinical trials, e.g., in Belgium and Switzerland [14,22]. The FDA (in June 2020) cancelled its earlier approval of the use of these agents as the EUA to treat adults and adolescents who weigh at least 50 kg and who are hospitalized with COVID-19 [69]. Later, in July, the HCQ arm of the international SOLIDARITY trial was halted as there is little or no reduction in mortality of hospitalized COVID-19 patients [34]. The cancellation was based on the lack of beneficial balance between clinical improvement and the risk of potential side effects. Findings of several recent studies have raised the dilemma about the utilization of HCQ and CQ in the treatment of COVID-19 as some results are supporting their use [70,71,72] and others are not [73,74,75,76], in terms of prescribing alone or in combinations. Importantly, WHO guideline does not recommend the use of these agents outside clinical trials [4].

HCQ/CQ may interact either with agents prescribed for chronic diseases (e.g., digoxin, amiodarone, etc.) or adopted for the management of COVID-19 in several guidelines (such as azithromycin and antiviral drugs) [77,78,79,80]. This effect might be continuous for many days as the half-life of the drug is long (more than 40 days) [77,80].

HCQ/CQ are not classified as a contraindication in pregnancy. Available information in pregnant patients show little [81], or no significant increase in the risk of congenital malformations or poor pregnancy outcomes in humans [82,83]. However, monitoring of the vital sign for the pregnant women is recommended.

### 4.1. Hydroxychloroquine

There are many criteria set by different national guidelines for the use of HCQ in COVID-19 patients. Prior to prescribing the HCQ, the severity of the COVID-19 symptoms like presence of dyspnea and patient general health status need to be considered. Generally, the drug is recommended for severe, confirmed, and hospitalized COVID-19 patients. However, India’s guideline distinctively recommends against the use of HCQ in severe cases [24]. The HCQ/CQ treatment options for the critically ill patients were removed from recent UAE guideline but kept for other COVID-19 patient under specific categories. Many treatment guidelines suspended the use of HCQ for managing COVID-19 cases [9,12]. However, other guidelines advice the use of HCQ/CQ in mild to moderate cases regardless of the hospitalization status [7,10,13,24,25]. In the UAE, HCQ can be used empirically in suspected symptomatic cases [13]. Furthermore, treatment guideline of India recommends it as a prophylactic option for the exposed healthcare workers and caregivers [24] unlike Pakistan’s guideline where it is discouraged [11]. In some guidelines, administration of the HCQ is conditioned on patient/guardian consent as per the WHO advice [7,13]. In Nigeria and Netherlands’ SWAB guidelines advised against the use of HCQ beyond clinical trials [19,33], while the other Dutch guideline (RIVM) recommended its use in severe or certain cases (such as oxygen saturation ≤ 93% on room air, on chronic oxygen supplementation, Age ≥ 60, BMI ≥ 30, diabetic (HbA1c ≥ 8), chronic heart disease/HTN, chronic lung disease, or immunocompromised) [20].

HCQ duration of treatment is usually five days, starting with a loading dose of 400 mg orally twice for one day and then a maintenance oral dose of 200 mg twice daily (or 400 mg once) for the following day 2 to 5. Depending on case severity, this duration can be extended to 7 or 10 days in the UAE guideline [13], 14 days in the Malaysian guideline [30], and up to 20 days if needed according to published Italian SITA/SIP physicians’ advice [17].

### 4.2. Chloroquine

Chloroquine (CQ) is the backbone of which the HCQ was derived from. Different studies claim that HCQ is more potent and safer than CQ [84,85,86]. The treatment dose of CQ as antimalarial is usually for three days with a total dose of 1500 mg [87]. In COVID-19 management, CQ was recommended as an alternative to HCQ in several guidelines [7,13,25]; however, it was not recommended in others (e.g., Italy, India, and Nigeria) [17,24,33].

In China and Pakistan, the 500 mg of CQ twice daily is advised to be given for 7 and 10 days, respectively [11,28]. In the UAE, the CQ dose duration depends on the severity of COVID-19 cases and is typically administered as 500 mg of CQ two times for first day then 250 mg once daily for 4 days as a safe option when ECG monitoring is unreachable [13]. Earlier Belgium guideline, before suspending its use along with HCQ, recommended 600 mg of CQ to be given once then 300 mg is given twice for 5 days [14].

### 4.3. Further Considerations in the Use of Antimalarial Agents in COVID-19 Patients

In general, the guidelines specify the dose of adult patients (>50 kg). However, little or no information is available on pediatric dosing. Treatment is, generally, not recommended for patients who are less than 12 years of age or weighing below 50 kg, while the treatment guidelines of Spain and the UAE have clearly stated doses for children in different weight groups [13,21].

Knowing that HCQ/CQ are metabolized in liver with some metabolites excreted by kidney [87,88]. Therefore, to avoid toxicity, clear recommendations and/or instructions for dose measurement in patients with kidney or liver failure should be illustrated. This issue can be further deteriorated by the possibility of COVID-19-induced liver and kidney damage [84,85,86,87,88]. In Belgium, it is clearly stated that the HCQ and CQ dosage adjustment needs to be considered on the basis of Glomerular filtration rate (GFR) [14]. The UAE consultation of antimalarial monographs for dosage adjustment and monitoring in liver or kidney failure is also promoted [13]. In Brazil, no further dosage adjustment is necessary except when the CrCl is below 15 mL/min [7].

There are many adverse effects affecting the heart, eyes, liver, and kidney; therefore, using these drugs should strictly be after patient examination [87]. Most available guidelines pointed out the side effect profile of HCQ/CQ especially the ventricular arrhythmias and the need for ECG monitoring.

Although HCQ and CQ are regarded safe during pregnancy, the treatment must be closely monitored, as mentioned above. In some guidelines, like in UAE, Belgium, and Italy, the safety of these agents was clearly noted [13,14,17]. Brazil and Indian guidelines marked HCQ/CQ as a contraindication in pregnancy [7,24]. In Pakistan, CQ only recommended in severe cases in pregnant women [11].

Several guidelines addressed the issue of the risk of drug-drug interaction of antimalarial agents with other drugs used in COVID-19 or drugs prescribed for chronic diseases. Belgian treatment guideline referred to the COVID-19 drug–drug interaction checker website which is created by Liverpool University for assessment of individual patient cases [14,89].

## 5. Systemic Corticosteroid

One of the main complications of COVID-19 or other severe respiratory infections is the elevated and uncontrolled inflammatory response with consequences ranging from mild dyspnea to ARDS, sepsis, and organ or multi-organ failure. Several inflammatory mediators (cytokines and interleukins) come into play in this pathological process [90]. Systemic corticosteroids (SCS) through their mechanism of action regulate the synthesis of proinflammatory factors and mitigate the destructive immune response. Despite exerting a useful therapeutic potential, corticosteroids may have a negative impact [91]. SCS therapy might enhance the viral load [92] and prolong the time for viral shedding. Several early versions of the guidelines were cautious in promoting SCS and limited their use to severe cases only (where ARDS is evident) and for the shortest period using the lowest effective dose [13,17,18,19,22,23,33].

In June 2020, the RECOVERY clinical trial findings outweighed the balance in preference toward corticosteroid therapy in patients requiring respiratory support. Treatment with dexamethasone (6 mg once daily for up to 10 days) caused significant reduction in deaths among COVID-19 patients on ventilators and those who received regular oxygen supplementation [93]. Another partially randomized study conducted on 85 COVID-19 patients concluded that methyl-prednisone reduced deaths, ICU admissions, and ventilator need [94]. Nonetheless, several retrospective studies found no protective effect of corticosteroids in COVID-19 patients and possibly harmful consequences in critical cases [95]. The timing (when), dosage (how much), and duration (for how long) of corticosteroid therapy are crucial to control the immune response but not to suppress it [96,97,98,99]. Furthermore, SCS therapy is only beneficial in patients requiring oxygen supplementation or in patients with ARDS, as evidence on its usefulness in mild cases is exceptionally low [93,100].

The corticosteroid therapy is associated with a highly unpleasant list of adverse effects including immunosuppression and might prolong viral shedding or bring about a secondary infection. However, a low dosage, equivalent to 0.5–1.0 mg/kg of methylprednisolone could be useful in avoiding these consequences [101,102,103]. Use of prophylactic antibiotics could be helpful in preventing any secondary infection [95]. On the use of corticosteroids for COVID-19, the Brazilian guideline permits a prior administration of the anthelmintic prophylaxis treatment to prevent incidental parasitic infection [7]. Short duration of treatment (10 days) with dexamethasone may also avert the incidence of other harmful effects such as hyperglycemia [95].

Antenatal corticosteroid administration is a common practice due to its importance in preventing premature labor. Its value is significant if the pregnancy is less than 32-week gestation (8 months) or less than 30 weeks for COVID-19 pregnant patients in the ICU setting [104]. Careful balance between benefit versus risk of corticosteroid therapy, on both COVID-19-positive mother and fetus, should be considered [105]. Note that 47% of COVID-19 hospitalized pregnant women appeared to result in preterm delivery [106]. WHO recommends the use of antenatal corticosteroids, from 24 to 34 weeks of gestation, if sufficient medical care is available for the newborn and no signs of maternal infection is present [4]. In USA, dexamethasone (or other alternatives if unavailable) is recommended in pregnant women with COVID-19 requiring all forms of supplemental oxygen [9]. The Society of Obstetricians and Gynecologists of Canada recommended low-dosage antenatal corticosteroid therapy with patient approval requirement [8]. In Saudi Arabia guideline, corticosteroids are indicated in severe to critical cases [12]. In case of pregnancy with COVID-19, dexamethasone should be substituted with prednisolone, 40 mg orally or IV, or hydrocortisone, 80 mg twice daily [12].

Corticosteroids are necessary in other chronic disorders such as COPD, asthma, and adrenal insufficiency which proved to be beneficial in some COVID-19 patients where they also present other chronic inflammatory disorders. Typically, the adrenal insufficiency disorder increases the risk of complications of COVID-19 due to diminished intrinsic corticosteroid activities. Doses of SCS could be increased to ameliorate the adrenal insufficiency that results from stress [107]. In Singapore, hypocortisolism is an indication for use of dexamethasone in the treatment of COVID-19 [29]. In UK, and earlier guidelines of USA, a low dose of corticosteroid can be used to manage other co-morbidities including asthma [9,23].

The most common corticosteroids used in COVID-19 treatment guidelines were dexamethasone and methylprednisolone. Hydrocortisone is safer and preferred in sepsis or septic shock [108]. However, no evidence of efficacy differences exists between these agents in case of sepsis [109]. The WHO recommends against the routine use of the corticosteroid therapy beyond clinical trials [4]. Warning and caution were mentioned in many treatment guidelines regarding use of SCS with COVID-19 cases. For example, Singapore treatment guideline recommended against the routine use of corticosteroids for COVID-19, apart from when its use is indicated including refractory shock and confirmed hypocortisolism [29]. Despite SCS promising results of RECOVERY trial, the Irish guideline in a recent update was cautious in recommending dexamethasone for COVID-19 cases and required prior discussion with consultants in a multidisciplinary setting [16]. In this context, the French guideline recommends not to use SCS in COVID-19 cases [15].

The use of SCS for patients with COVID-19 is the last choice treatment as per the Indian guideline [24]. In an earlier Irish COVID-19 treatment guideline, the low SCS doses were used in severe cases with multi-organ failure when there was no response to immunosuppressant drugs [16]. In the Pakistan guideline, all patients requiring oxygen should be on steroids (dexamethasone or methylprednisolone) as well [11]. The choice of steroid usage is at the discretion of the clinician. In addition, Singapore and Switzerland COVID-19 treatment guidelines both considered SCS in patients who have severe inflammation for more than seven days [22,29]. The Italian guideline advised its use in ARDS for up to 10 days [17].

### 5.1. Dexamethasone

Dexamethasone is a long-acting SCS with biological half-life of 36 to 72 h and a potency about 25 times greater than the short-acting hydrocortisone [110]. Its major importance in COVID-19 is attributed to the preliminary findings of the RECOVERY trial published in July 2020. The 6 mg of dexamethasone dose per day for 10 days can reduce mortality rate in COVID-19 patients requiring oxygen supplementation or on mechanical ventilation [93].

New updates of several national guidelines have considered these RECOVERY trial results [11,12,16,18,19,22,23,29,31]. Switzerland, Malaysia, and Singapore consider COVID-19 symptoms for more than 7 days as another criterion in prescribing dexamethasone [22,29,30]. Switzerland also considered severe inflammation and the rapidly deteriorating disease course in the indication of dexamethasone for COVID-19 cases [22].

### 5.2. Methylprednisolone

Methylprednisolone is an intermediate-acting steroid (biological half-life: 12 to 36 h) and it is 5 times more potent than hydrocortisone [110]. In June 2020, results of a randomized multicenter trial (GLUCOCOVID) were published (pre-print) showing that a 6-day regimen of methylprednisolone can reduce mortality, ICU admission, and the need for ventilation. The dosage used is as follows: 40 mg/12 h for 3 days, then 20 mg/12 h for 3 days [94]. Other findings also suggested that methylprednisolone improved COVID-19 patients’ outcomes when given as an IV at a dose of 0.5–1.0 mg/kg for 3 days in non-ICU patients and 5–7 days in ICU-admitted patients [94]. This later evidence was applied in the updated UAE COVID-19 treatment guideline as well [13].

This drug was recommended in many treatment guidelines [9,11,13,20,24,28,30]. In Netherland RIVM treatment guideline, the recommended methylprednisolone dose is 40 mg (0.5 mg/kg/day) three times daily for up to 7 days [20]. In Singapore, 32 mg daily dose of methylprednisolone is recommended [29], while in India 0.5–1.0 mg/kg/day for 3–5 days or more in moderate cases with increasing oxygenation need [24]. In pediatric COVID-19 cases in Saudi Arabia and Pakistan, the criterion for increased treatment duration is prolonged hypoxia [11,12]. A higher dosage of 1 to 2 mg/kg/day for 3–7 days was recommended in severe cases in India and China [24,28].

### 5.3. Prednisone

Prednisone is considered in USA, South Africa, and Singapore COVID-19 treatment guidelines as an alternative to dexamethasone [18,23,60]. This drug is available only as an oral dosage form. It is intermediate-acting corticosteroid, with biological half-life 12 to 36 h [110].

### 5.4. Prednisolone

The potency of prednisolone is similar to prednisone with a comparable biological half-life [110]. It is only recommended as an option in pediatric COVID-19 patients at a dose of 1 mg/kg once daily as per Saudi Arabia treatment guideline [12].

### 5.5. Hydrocortisone

Hydrocortisone is a short-acting corticosteroid with biological half-life of 8 to 12 h, therefore it is more frequently dosed (2–4 times daily) [110]. Treatment of COVID-19 with low dose of hydrocortisone was advised in the treatment guidelines of Egypt, and in USA as an alternative glucocorticoid if dexamethasone is not available [9,10].

## 6. Immune-Based Therapy

Agents that modulate the immune response are being explored as adjunctive treatments for the management of moderate to severe COVID-19 cases. These agents include interleukin-2, alpha-interferon, gamma-interferon, and monoclonal antibodies [111]. The immune system potency is essential in fighting infection; nevertheless, an aggressive and aggravated immune response with fatal consequences accompanies the late stages of COVID-19 infection to prevent or treat ARDS, cytokine storm, and organ(s) failure. Seven of these medications were used in managing COVID-19 cases with no evidence of such activity existing yet. These medications are undergoing clinical trials to assess their efficacy against the coronavirus [112].

### 6.1. Tocilizumab

Tocilizumab is a recombinant monoclonal antibody of IgG1 class, interleukin-6 receptor inhibitor [113]. The TESEO cohort and EMPACTA studies showed tocilizumab’s role in reducing the risk of invasive mechanical ventilation or death in patients with severe COVID-19 pneumonia [114,115]. While the COVACTA trial opposed these results and concluded no improvement of the clinical status nor mortality reduction in pneumonia-associated COVID-19 patients [116].

According to the Swiss guideline, tocilizumab is considered as an antiviral and an immunomodulatory treatment in both early and later stage of COVID-19 infection along with being an essential drug in the cytokine storm [22]. The Chinese Pharmaceutical Association, Chinese National Health Commission, and the UAE guideline recommend its usage in patients with extensive lesions in both lungs and/or elevated IL-6 levels [13,27,28]. Furthermore, the Spanish guideline recommends using this drug for patients with inflammatory cascade leading to effecting patients’ ventilation [21]. Many guidelines recommended tocilizumab as an off-label medication for patients with cytokine release syndrome [12,13,16,18,24,30]. RIVM of the Netherlands recommends usage of this drug for non-severe COVID-19 patients and in severe COVID-19 patients suffering from a respiratory failure or are under mechanical ventilation [20]. Pakistan’s guideline also permits tocilizumab usage to patients with moderate to severe cases of COVID-19 with ARDS or severe life-threatening cytokine release syndrome [11]. UAE provides this drug to ICU COVID-19 patients suffering from severe cytokine release syndrome [13]. Singapore does not recommend the use of tocilizumab, but would consider it in the case of cytokine storm or hyper inflammatory patients [29]. USA and Nigeria recommend against the use of tocilizumab for COVID-19 unless on a clinical trial [9,33].

This medication is normally given as a starting dose of 4 mg/kg every 4 weeks followed by an increase to 8 mg/kg every 4 weeks based on clinical response according to the Chinese guidelines [27,28]. The Irish guideline allows the use of tocilizumab as 400 mg dissolved in 100 mL of normal saline and set on a continuous infusion for more than 1 h and an additional dose is recommended within 12 h for poor response of the first dose [16]. As per Saudi Arabia and UAE protocols, the maximum single tocilizumab dose of 800 mg is recommended over a two-time interval [12,13], while the Pakistani guideline urge to repeat the tocilizumab dose once only [11]. The tocilizumab dosing in Switzerland and Netherlands guidelines is considered as 8 mg/kg, and the maximum single tocilizumab dose is 800 mg with the potential use of a second dose when needed [20,22]. If partial or incomplete clinical response is present, a third dose can be considered according to Italy’s guideline [18]. Spain’s adult tocilizumab dosing regimen is based on patients weighing ≥75 kg: 600 mg single dose and patients weighing <75 kg: single dose of 400 mg and a second infusion can be given after 12 h of the first infusion dose in patients experiencing a rebound in analytical parameters after partial improvement [21]. 

In Spain, Saudi Arabia, and the UAE, the Tocilizumab pediatric dosing regimen for children weighing < 30 kg is 12 mg/kg intravenously and 8 mg/kg intravenously when the weight is ≥30 kg, although the 8 mg/kg/iv protocol was applied to all COVID-19 children in these countries [12,13,21].

Tocilizumab is contraindicated in uncontrolled bacterial and fungal infections as indicated in the Swiss COVID-19 treatment guideline [22], or active infections such as tuberculosis as highlighted by many other guidelines [11,12,13,16,27,28]. This drug is not recommended as a COVID-19 treatment option in the cases of (i) aspartate transaminase/alanine aminotransferase (AST/ALT) values greater than 5 [13] or 10 [21] times of its normal upper limit, (ii) neutrophils < 500 cells/mmc, (ii) platelets < 50,000 cells/mmc, (iv) recognized sepsis by pathogens other than SARS-CoV-2, (v) presence of comorbidity that can lead to a poor prognosis, (vi) multiple organ failure, (vii) complicated diverticulitis or intestinal perforation, or (vii) ongoing skin infection [11,12,13,16,29]. Contraindications of the medication include the concomitant use of anti-TNF agents, disease-modifying antirheumatic drugs (DMARDs), live vaccines, and other immunosuppressive therapy should also be avoided [12].

Italian and Pakistani guidelines advised a prior risk and benefit assessment for COVID-19 pregnant women [11,18], while Saudi Arabia and the UAE simply considered this as one of its contraindication cases [12,13].

### 6.2. Siltuximab

Siltuximab is an interleukin-6 inhibitor and monoclonal antibody [117]. In a cohort study, siltuximab proved to be beneficial in lowering mortality and cytokine-driven hyper-inflammation which is associated with rapidly progressing respiratory failure in COVID-19 patients who need ventilator support [117].

Spain is the only country administering siltuximab as a compassionate treatment in interstitial pneumonia with severe respiratory failure, rapid respiratory worsening requiring noninvasive or invasive ventilation, presence of extra pulmonary organ failure, or severe systemic inflammatory response [21]. In the USA, it is not recommended for COVID-19 patients unless in a clinical trial setting [9].

Siltuximab use is not recommended in COVID-19 patients with HIV, human herpes virus, Epstein–Barr virus, tuberculosis, neutrophils ≤ 1.0 × 109/L, platelets ≤ 50 × 109/L, AST/ALT values more than 5 times of its normal upper limit, and total bilirubin ≥ 2.0 times its normal upper limit [21]. Siltuximab is also not recommended for pregnant or breastfeeding women [9,21].

### 6.3. Sarilumab

Sarilumab is an IL-6 inhibitor and is recommended only in Spain guideline with a recommended dose 200 or 400 mg single infusion, although no evidence on its efficacy is yet proven [21]. A cohort study conducted on COVID-19 severe pneumonia and systemic hyper-inflammation patients who received sarilumab resulted in no difference in the overall clinical improvement and mortality when compared to the standard care, although showed faster recovery in patients with minor lung consolidation at baseline [118]. Safety and efficacy of pediatric dosage is not available for this drug.

### 6.4. Canakinumab

Canakinumab is human anti-IL-1β monoclonal antibody which works to neutralize 1β signaling [119]. One case was reported on an elderly COVID-19 patient with ARDS who used canakinumab and resulted in significant reduction in high IL-6 levels and NK cells expressing CD56bright (associated with cytokine release) [120].

Italy is the only country considering canakinumab as a compassionate treatment through Novartis’ Managed Access Program when no other treatment is available and complication from COVID-19 is serious or life-threatening [17]. Dosing of canakinumab is 600 mg in 250 mL of 5% dextrose intravenously over 2 h for patients weighing 40–60 kg (not exceeding 10 mg/kg) [17].

### 6.5. Anakinra

Anakinra is an interleukin antagonist. In an Italian cohort study, the use of a high dose of anakinra on patients with COVID-19 and ARDS resulted in clinical improvements in 72% of patients [121]. Another study conducted as case series proceeded to prove anakinra as beneficiary in preventing the need for mechanical ventilation on COVID-19 patients with an onset of acute hypoxemic respiratory failure (AHRF) and cytokine storm syndrome (CSS) [122]. Only UAE guideline considered anakinra as an alternative to tocilizumab when it is contraindicated [13].

Anakinra therapy should not be initiated in patients with neutropenia and is not recommended for AST/ALT values greater than 1.5–fold of its normal upper limit [123].

### 6.6. Ruxolitinib

Until December 2020, no evidence exists proving the efficacy of ruxolitinib in reducing mortality or morbidity of COVID-19 patients. The ruxolitinib compassionate use in Spain is for COVID-19 patients with interstitial pneumonia, severe respiratory failure, rapid respiratory worsening requiring noninvasive or invasive ventilation, and extra pulmonary organ failure [21]. Furthermore, Italy considers this medication for COVID-19 cases through compassionate use of Novartis’ Managed Access program [17].

In Spain, the ruxolitinib dose for a COVID-19 case is 5 mg twice daily for 14 days [21]. In Italy, age groups of 6–12 years old and above 12 years old will have doses of 5 mg once daily and twice daily, alternately, for 7 days [17].

Ruxolitinib is not recommended for COVID-19 cases when the neutrophils level is <500 cells/mmc, platelets level is <50,000 cells/mmc, hemoglobin level is <80 g/L (PV), sepsis, or in treatment with fluconazole (>200 mg/day) [21]. Ruxolitinib is also not recommended in pregnant or breastfeeding women and in people with severe renal impairment [17,21].

### 6.7. Baricitinib

Baricitinib is a selective and reversible Janus kinase 1 and 2 inhibitors. An observational longitudinal clinical study conducted on COVID-19 pneumonia patients administered baricitinib and resulted in prevention of viral progression and increased antibody production against SARS58 CoV-2 spike protein [124]. The USA guideline does not recommend the use of baricitinib unless during a clinical trial [9].

The adult baricitinib dose as per Spain guideline is 4 mg once a day for 7–14 days and for patients ≥ 75 years of age, a dose of 2 mg once daily is permitted [21]. The pediatric dose safety has not been established yet.

This drug is not recommended to be used in the cases of lymphocytes < 200 cells/mmc, neutrophils < 500 cells/mmc, platelets < 50,000 cells/mmc, hemoglobin < 8 g/dL, AST/ALT values greater than 5 times of its normal upper limit, creatinine clearance ≤ 30 mL/min, sepsis, liver failure, and tuberculosis [21]. The contraindication includes pregnancy [21].

## 7. Interferon Type I: IFN Type I

Interferon (IFN) type I (including IFN alpha and IFN beta) fits in both therapeutic categories of antiviral and immune modulators. These agents are signaling proteins that augment host viral defense upon release by virus-infected cells. Later in the disease process, IFN type I may regulate the immune response including levels of IL-6 [125].

The clinical importance of immune-regulating effect of IFN in COVID-19 is controversial. One study indicated that deficiency of interferon couples with severe forms of the disease and IFN levels inversely correlate with viral load [126,127]. However, late administration of IFN-α or β (i.e., more than 10 days since COVID-19 diagnosis) resulted in poor prognosis of the disease in contrast to early therapy suggesting pathologic effect at advanced, inflammatory stage of COVID-19 [127,128]. The early use of IFN increases recovery and lowers mortality in COVID-19 patients [127,129]. This beneficial effect of IFN is attributed to its antiviral activity, highlighting the significance of timing in IFN administration. Additionally, the prophylactic use of IFN offered protection for health care workers [130]. IFN type III retains antiviral activity of INF type I but not the immune-modulation and could be a potential treatment strategy that suppresses viral load without inducing a harmful hyper-inflammation [131,132]. Combinations with other antivirals provide favorable outcomes according to Chinese study [133].

Treatment guidelines in USA, Canada, Netherlands (SWAB), and Nigeria do not recommend the use of interferon for COVID-19 unless in a clinical trial setting [8,9,20,33]. Guidelines in Saudi Arabia and the UAE recommend the use of triple therapy of interferon IFN (IFN beta 1b: dosage of 8 MIU on alternate days for 3 doses) with ribavirin and lopinavir/ritonavir in severe and critically ill COVID-19 patients [12,13]. The Chinese guideline considered the use of interferon alpha 1b (5 MIU twice daily) alone or in combinations with antivirals such as ribavirin and lopinavir/ritonavir [28]. In the UAE, interferon alpha or beta (beta-interferon 1b: 0.25 mg on alternative days for total of 5–7 doses or Pegylated interferon-Alfa 2a:180 mcg weekly for 2 weeks) could be considered in triple combination in severe and critical cases or as an add-on treatment in case-by-case basis in moderate cases [13]. To reach its antiviral activity, interferon beta 1a (administered as 44 mcg stat then every other day) or interferon beta 1b (administered as 250 mcg stat then every other day) are given over 3–5 doses in addition to antivirals in Malaysian guideline [30].

Chinese guidelines considered the pulmonary route of admission for IFN type I [28] while subcutaneous administration is recommended in Singapore, Malaysia, UAE, and Saudi Arabia [12,13,29,30]. Both Singapore and Saudi Arabia guidelines promote the early use of interferon to prevent its unfavorable elate effect (within 6–7 days of COVID-19 symptom appearance) [12,29]. In Singapore, the interferon therapy may continue for no more than 7 days to avoid interacting with hyper-inflammatory stage of illness; however, it is recommended for 14 days in Saudi Arabia [12,29].

## 8. Antibiotic Therapy

Antibiotics are used in the treatment of upper respiratory tract infections depending on the severity of the infection and the patient health status [134]. However, the use of antibiotics is not part of any viral infection treatment protocols unless it is associated with secondary bacterial infection [135]. Many guidelines do not recommend the use of antibiotics in mild cases (treatment or prophylaxis) or outside hospitals [9,14,16,21,33]. Guidelines indicate antibiotics should only be used if there is a clinical suspicion in moderate or severe cases. The empiric therapy for all possible pathogens is recommended to be started early with de-escalation protocols if the culture transpires to be negative [136].

During the early days of this pandemic, physicians were looking for treatment options and the use of azithromycin in the management of COVID-19 pandemic appeared in open-label non-randomized trial in France [67]. That article opened the door for a long debate around the role of azithromycin in the treatment of COVID-19 patients. Some of the articles argued that azithromycin does not only have antimicrobial activity, but presents antiviral and immunomodulation properties against different types of viruses, making it a potential treatment option for COVID-19 virus [137]. On the opposite side, articles highlighted these properties are weak and the evidence on such activities was not based on COVID-19 patients [138,139].

In severe COVID-19 cases, early empirical antibiotics such as amoxicillin/clavulanate, or third-generation cephalosporins (ceftriaxone, cefotaxime, or ceftaroline), in combination with azithromycin were recommended in the treatment guidelines of Malaysia and France [15,30].

Regarding azithromycin, the WHO did not include the use of this antibiotic as a treatment option within its guidelines [4]. In many guidelines, the use of azithromycin with HCQ is not recommended due to the associated adverse effects observed with this treatment strategy [9,15,16,17,19], while Nigeria’s guideline did not recommend it unless in a clinical trial setting [33]. Other countries like Thailand, and Brazil enabled the use of azithromycin in combination with HCQ in severely ill patients [7,25].

The WHO recommendation was against the use of antibiotics in mild cases and prescriptions should be dispensed when doubts in moderate cases are strong [4]. The recommendation was to use empiric therapy in severe cases and de-escalate antibiotic use in negative cultures [140]. The Chinese authorities insisted on the rational use of antibiotics in managing COVID-19 cases [28], while the UAE and India guidelines limited the antibiotics use for COVID-19 cases with severe acute respiratory illness (SARI) or sepsis [13,24]. Pakistani authorities linked the use of antibiotics to white blood cells count when an infection is suspected [11]. Linezolid or vancomycin was recommended in France, the UK, and Egypt treatment guidelines [10,15,23]. Clindamycin should be added if streptococcal/staphylococcal toxic shock syndrome is suspected as per the Malaysian guideline [30].

France and UK guidelines provided detailed antibiotic treatment options based on evidence from clinical studies on animals [15,23]. These guidelines recommend the use of doxycycline in several cases instead of amoxicillin because it has a broader spectrum, particularly against mycoplasma pneumonia, which are more likely to be secondary bacterial causes of pneumonia during the COVID-19 pandemic. The COVID-19 guidelines of Belgium, Brazil, Canada, Ireland, Spain, and USA do not recommend antibiotic prophylactic use, while empiric treatment is considered in severe cases and depend on the comorbidity, patient situation, and local epidemiology with de-escalation of antibiotic therapy in absence of MRSA infections [7,8,9,14,16,21].

## 9. Anticoagulants

One of the causes of COVID-19 mortality is abnormal hypercoagulation along with an increased D-dimer and fibrin degradation product (FDP) [141,142,143]. Furthermore, when the patient is on a longer bed rest, the chance of venous thromboembolism (VTE) is higher. In a retrospective study preformed in China on severe COVID-19 patients treated with the low molecular weight heparin (LMWH) resulted in better prognosis in meeting sepsis-induced coagulopathy (SIC) with increased D-dimer [144], while in a multicenter prospective cohort study, severe COVID-19 patients with secondary ARDS who were on anticoagulation therapy still experienced life-threatening thrombotic complications [145].

The USA guideline encourages patients on long-term use of anticoagulant or antiplatelet therapy to continue using their medication even with a COVID-19 diagnosis but does not recommend it as prevention for non-hospitalized patients with COVID-19 [9]. As for adult hospitalized patients with COVID-19, VTE prophylactic treatment should be given per the standard care but patients should not be discharged with a prophylactic long-term use [9].

In the guidelines of UAE, South Africa, Egypt, and Netherlands, all admitted COVID-19 patients are subject to receive prophylactic anticoagulant for VTE whether they are at risk or not if no contraindication is present [13,19,32]. The Singapore treatment guideline recommends VTE prophylaxis treatment for only critically ill patients and those with severe diseases [29]. In Egypt, the treatment recommends to give enoxaparin for recumbency patients as well as prophylactic for those show signs of pulmonary embolism [10]. 

The Belgium guidance recommends for patients on chronic oral anticoagulants to switch to LMWH to avoid interactions with many drugs used to treat patients with COVID-19 infection [14].

LMWH is recommended by India, KSA, Belgium, and Chinese pharmaceutical Association guidelines [12,14,24,27], while unfractionated heparin is used in guidelines of Netherlands, India, and Ireland at prophylactic and therapeutic doses [16,19,24]. Unfractionated heparin is preferred in case of renal insufficiency in Malaysian guideline [30]. Dalteparin, calcium nadroparin, and fondaparinux sodium are used by the Netherlands [19]. Enoxaparin is recommended in many guidelines [11,12,16,19,30]. Moreover, tinzaparin is recommended in Ireland guideline [16].

In South Africa, therapeutic doses should be given if D-dimer > 6 times its upper limit or if the patient case requires supplemental oxygen at more than 60 % oxygen concentration [32].

For women with COVID-19 undertaking labor, anticoagulation therapy should be administered as per the standard care [9,12]. Unfractionated heparin and LMWH are safe in breastfeeding while direct-acting oral anticoagulants are not [9].

## 10. Other Medications

Some medications, remedies, and vitamins have been mentioned in several guidelines [9,10,13,16,19,27,33].

### 10.1. Non-Steroidal Anti-Inflammatory Drugs (NSAIDs)

USA and Ireland guidelines advise patients who are already on NSAID therapy to continue taking their medications when contracting COVID-19 [9,16].

### 10.2. Zinc

It is not recommended in Netherlands for COVID-19 patients to start zinc supplementation [19]. However, the UAE recommends taking 100 mg of elemental zinc daily for COVID-19 patients or as a prophylaxis strategy [13].

### 10.3. Vitamin C

In the UAE, it is recommended to take vitamin C (orally or IV, 1–3 g daily) [13]. China’s Pharmaceutical Association guideline recommends administering 100 to 200 mg per kilogram intravenously per day for COVID-19 patients until the oxygenation index improves significantly [27]. In Egypt guideline recommends to take ascorbic acid 500 mg every 12 h for all cases [10].

### 10.4. Vitamin D

There are no data available on prevention or treatment of COVID-19 according to the Netherlands’ guideline [19].

### 10.5. Multivitamin

The Nigerian guideline was open to administering multivitamins and mineral supplements [33]. 

## 11. COVID-19 Managements for Special Population

Patients with glucose-6-phosphate-dehydrogenase (G6PD) deficiency are not recommended to use HCQ/CQ due to the risk of hemolytic anemia [21].

USA’s NIH recommends COVID-19 treatment in HIV-positive patients to be the same as the general population. The recommendation is to, also, continue with antiretroviral therapy and prophylaxis for opportunistic infections whenever possible in COVID-91 patients with HIV who infected by COVID-19 virus, including in those who require hospitalization [9].

USA and Saudi Arabia guidelines recommend consulting a transplant specialist before adjusting immunosuppressive medications of transplant and cellular therapy patients who have COVID-19 infection and to monitor drug–drug interactions or toxicities regarding other concomitant immunosuppressant, prophylactic antimicrobials, or other medications [9,12]. The Netherlands’ guideline recommends HCQ for 5 days in immunosuppressed patients hospitalized with non-severe COVID-19 who are on cancer treatment for a 1 year, or using immunosuppressive drugs, had an organ transplant, or a bone marrow transplant, HIV/AIDS patients, leukemia patients, lymphoma patients, have SLE, or vasculitis, and if oxygen requirement is ≥ 2 L and hs-CRP > 70. Then, tocilizumab should be considered as a second line [19].

For cancer patients, the NIH guideline recommends molecular diagnostic testing to be performed for severe COVID-19 patients. Otherwise, the treatment is the same as the general population [9].

Influenza and COVID-19 coinfections are possible. The treatment for both is quite similar starting with an empirical therapy using oseltamivir even if the result of influenza test was not confirmed yet. Once influenza is ruled out, the antiviral treatment can be stopped [9].

## 12. Strength and Limitation and of This Review Article

Many COVID-19 treatment guidelines and protocols (n = 30) were screened. All official guidelines published were included in this review article to provide a comprehensive global image of current approaches in COVID-19 treatment that can be used by practitioners worldwide. The included guidelines covered countries from the six regions of the world and were published in English language or been translated using Google translator. The accuracy of translation of guidelines were validated by all authors. However, many other COVID-19 treatment guidelines were excluded due to difficulty in interpreting the translated versions (Table 1).

## 13. Conclusions

COVID-19 is a pandemic with a rapidly increasing incidence of infections and deaths. Many pharmacologic therapies are being used, off-label, or considered for treatment worldwide in responses to the pandemic. Through searching 27 treatment guidelines from 23 countries and three references guidelines that have been published in 2020, various drugs were recommended for treating patients with COVID-19 infection. However, there is no strong evidence on the effectiveness and safety of most medications that are been used in treating patients with of COVID-19 infection over the year.

There are four main classes of drugs recommended in the treatment guidelines worldwide: antiviral drugs (eight drugs), antimalarial drugs (two drugs), systematic corticosteroids (five drugs), and immune-based therapy (seven drugs). In addition, there are many other groups to eradicate concurrent infection and prevent complications. These drug classes were listed in most of the guidelines, either with compressive recommendations on the use of some of these drugs or with restrictions for use in clinical trials settings only. Generally, there is a big variation between these guidelines. These mainly related to indication of the drugs, types of drugs, dosage regimen, period of treatment, and safety of use in different groups of patients. However, in all of the guidelines, the recommendations for the treatment with these drugs based on severity of case and health condition of patient.

Due to the novelty of the disease and the great pressure on healthcare, many issues are still without clear answers. This is mostly related to the low number of publications on the effectiveness and safety of these drugs in COVID-19 patients. Furthermore, the impact of high costs of some of the proposed treatment regimens on communities with limited financial resources is still unknown.

## Figures and Tables

**Table 1:** Summary of medications used for managing COVID-19 cases as per 24 treatment guidelines.

	Medications																							
	Anti-Virial Drugs							Anti-Malarial Drugs		Systematic Corticosteroids					Immune-Based Therapy							Interferon Type I: IFN Type I	Antibiotic Therapy	Anti-Coagulant
**Region**	**Remdesivir**	**Lopinavir /Ritonavir**	**Darunavir/Cobicistat **	**Favipiravir**	**Umifenovir**	**Ribavirin**	**Oseltamivir**	**Hydroxychloroquine**	**Chloroquine**	**Dexamethasone**	**Methylprednisolone**	**Prednisone**	**Prednisolone**	**Hydrocortisone**	**Tocilizumab**	**Siltuximab**	**Sarilumab**	**Canakinumab**	**Anakinra**	**Ruxolitinib**	**Baricitinib**			
**Reference Guidelines**																								
**WHO [4]**																							✓	✓
**FDA [5]**	✓							✕	✕												✓			
**EMA [6]**	✓									✓														
**Americas**																								
Brazil [7]	✓							✓	✓	✓													✓	
Canada [8]								✕		✓												✕	✓	
USA [9]	✓	✕						✕	✕	✓	✓	✓		✓	✓	✓					✓	✕	✓	✓
**Eastern Mediterranean**																								
Egypt [10]		✓					✓	✓						✓									✓	✓
Pakistan [11]	✓							✕	✕	✓	✓				✓								✓	✓
Saudi Arabia [12]	✓	✓		✓		✓				✓	✓		✓		✓							✓		✓
United Arab Emirates [13]	✓			✓		✓		✓	✓	✓	✓				✓				✓			✓	✓	✓
**Europe**																								
Belgium [14]	✓	✓						✕	✕	✓													✓	✓
France [15]										✕													✓	
Ireland [16]		✓						✕		✓					✓								✓	✓
Italy [17,18]	✓	✕	✕					✓	✕	✓					✓			✓		✓			✓	
Netherlands [19,20]								✓	✓	✓	✓				✓							✕	✓	✓
Spain [21]	✓							✓	✓						✓	✓	✓			✓	✓		✓	
Switzerland [22]								✕	✕	✓					✓									
United Kingdom [23]																							✓	
**South-East Asia**																								
India [24]	✓							✕	✕	✓	✓				✓								✓	✓
Thailand [25]		✓		✓				✓	✓														✓	
**Western Pacific**																								
China [26,27,28]		✓	✓		✓	✓			✓		✓				✓							✓	✓	
Singapore [29]	✓	✓				✓		✕	✕	✓		✓			✓							✓		✓
Malaysia [30]				✓				✓		✓	✓				✓							✓	✓	✓
Australia [31]	✓							✕	✕	✓														
**Africa**																								
South Africa [32]								✕	✕	✓		✓												✓
Nigeria [33]	✕	✕		✕	✕			✕	✕						✕							✕	✕	

✓ the use of these agents in the treatment of COVID-19 is recommended; ✕ the use of these agents in the treatment of COVID-19 is not recommended.

## Data Availability

Not applicable.
